# Adult diffuse gliomas produce mRNA transcripts encoding EGFR isoforms lacking a tyrosine kinase domain

**DOI:** 10.3892/ijo.2011.1287

**Published:** 2011-12-08

**Authors:** ANGÉLIQUE GUILLAUDEAU, KARINE DURAND, HÉLÈNE RABINOVITCH-CHABLE, ISABELLE POMMEPUY, LAURA MESTUROUX, SANDRINE ROBERT, ALAIN CHAUNAVEL, JEAN-JACQUES MOREAU, FRANÇOIS LABROUSSE

**Affiliations:** 1 Department of Pathology, Dupuytren University Hospital, 2 Avenue Martin Luther King, F-87042 Limoges, France; 2 Department of Biochemistry and Molecular Genetics, Dupuytren University Hospital, 2 Avenue Martin Luther King, F-87042 Limoges, France; 3 Department of Neurosurgery, Dupuytren University Hospital, 2 Avenue Martin Luther King, F-87042 Limoges, France

**Keywords:** gliomas, glioblastoma, epidermal growth factor receptor, EGFR isoforms, *EGFR* mRNA variants

## Abstract

The epidermal growth factor receptor (*EGFR*) gene encodes four alternatively spliced mRNA, variants 1, 2, 3 and 4, respectively, encoding the whole isoform a (EGFR) and truncated isoforms b, c and d, all of which lack the receptor’s intracellular domain. In addition, a mutant EGFRvIII differs from isoform a in a truncated extracellular domain. The expression pattern of these isoforms is unknown in adult diffuse gliomas. Thus, we investigated in 47 cases: i) EGFR protein expression by immunohistochemistry using an extracellular domain-recognizing antibody (Ext-Ab) and an intracellular domain specific one (Int-Ab), ii) mRNA expression of EGFRv1, -v2, -v3, -v4 and -vIII by RT-PCR and iii) *EGFR* amplification by fluorescent *in situ* hybridization. The relation of these data with histological criteria and patient outcome was studied. The immunostaining was stronger with the Ext-Ab than with the Int-Ab. EGFRv1, -v2, -v3 and -v4 mRNA expression were highly correlated. They were expressed in all tumors, with highest levels in glioblastomas. EGFRv1 strong levels and the presence of vIII mRNAs were more closely associated with Int-Ab staining. *EGFR* gene amplification concerned only glioblastomas and was associated with the presence of EGFRvIII and high levels of EGFRv2, -v3 and -v4 transcripts. A pejorative outcome was associated with: histology (glioblastomas), *EGFR* amplification, strong Int-Ab labeling and high levels of variant mRNAs. Our results indicated that the full-length EGFR and mutant EGFRvIII are not the sole EGFR isoform expressed in diffuse gliomas. This could explain discordant immunohistochemical results reported in the literature and may have therapeutic implications.

## Introduction

According to the World Health Organization classification (WHO), adult diffuse gliomas include astrocytomas, glioblastomas, oligodendrogliomas and oligoastrocytomas (1). Despite therapeutic advances, these tumors remain incurable and the prognosis for patients afflicted with anaplastic tumors and glioblastomas is still very poor (2).

The epidermal growth factor receptor (EGFR) belongs to HER family, a group of four receptors. Many reports have shown that EGFR contribute to diffuse glioma oncogenesis (3–8). The most prevalent EGFR pathway modifications involved are *EGFR* gene amplification and receptor overexpression, the latter of which remains controversial (9–15).

The *EGFR* gene is located in 7p12 and generates a first type of mRNA transcript, referred to as EGFR variant 1 (EGFRv1), which encodes the full-length receptor, known as isoform a, EGFR or HER1. Isoform a is a transmembrane protein with an intracellular tyrosine kinase domain. Ligand binding on the extracellular domain leads to stimulation of cellular proliferation and cell survival pathways. Glioblastomas harboring *EGFR* gene amplification frequently contain a genetic variant, EGFRvIII, which encodes a mutant receptor with an altered extracellular domain that renders it constitutively active (16–18).

In addition to variant v1, the *EGFR* gene generates three other alternatively spliced transcripts, referred to as variants 2–4 (EGFRv2-v4). The corresponding mRNAs are shorter than the EGFRv1 transcript and, respectively, encode truncated forms of the receptor (isoforms b, c and d), which lack the cytoplasmic tyrosine kinase domain. Soluble isoforms have been reported (19–21). The role of these truncated EGFR isoforms remains poorly known. *In vitro*, they have been shown to decrease cellular proliferation (22,23). The hypotheses for this cellular growth inhibition include the competitive binding of EGFR ligands by the truncated isoforms (24,25) and formation of catalytically inactive heterodimers of different isoforms, which interfere with the formation of functional holoreceptor dimers. This consequently prevents intracellular kinase activity (26). To our knowledge, expressions of truncated EGFR isoforms and their transcripts have never been studied in gliomas.

To assess whether EGFR protein isoforms and their corresponding transcripts are expressed in diffuse gliomas, we performed an immunohistochemical analysis and determined the expression patterns of EGFRv1, -v2, -v3, -v4 and mutant EGFRvIII mRNAs. Results were analyzed with respect to the clinical data, patient outcome, histological tumor type and presence or absence of *EGFR* gene amplification.

## Patients and methods

### Patients and tissue samples

Tumors were obtained from 47 adult patients diagnosed with infiltrating glioma who were undergoing surgery at Limoges Dupuytren University Hospital between 1995 and 2011. Clinical and survival data were obtained by a retrospective query and all samples were used in accordance with French bioethics laws regarding patient information and con-sent. No patients received EGFR-targeted therapeutics. Tumor samples were fixed in 4% formalin at the time of resection. They were then embedded in paraffin and tumor sections were stained with hemalum phloxine saffron. Part of the surgical specimen was snap-frozen and conserved at −80°C. The histological type and grade of gliomas were determined according to the WHO classification (1). Non-tumor tissue was used as a control.

### Immunohistochemistry

**Figure 1:**
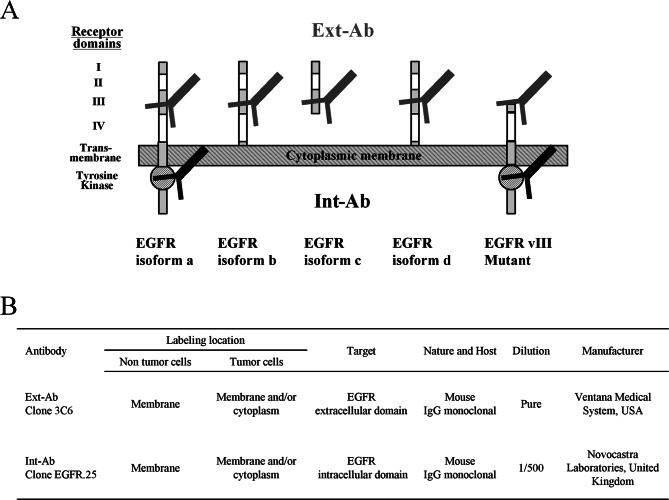
Characteristics of antibodies used in immunohistochemistry. (A), Ext-Ab (grey) and Int-Ab (black) targeting different domains of EGFR isoforms a, b, c, d and the EGFRvIII mutant; (B), Ext-Ab and Int-Ab characteristics.

Sections (5 μm) were cut from paraffin-embedded tumors and stained with two different anti-EGFR antibodies (Fig. 1). One antibody targeted the extracellular domain (Ext-Ab) and the other intracellular domain (Int-Ab). Sample slides were processed automatically (BenchMark XT ICH/ISH, Ventana Medical Systems, Tucson, AZ, USA) according to protocols supplied by the manufacturers. EGFR staining was quantified according to a semiquantitative method proposed by Hirsch *et al* (27), as previously described (28). Staining was scored for labeling intensity (1, negative or trace; 2, weak; 3, moderate; 4, intense), percentage of positive cells per slide (0%–100%) and for the Hirsch score resulting from multiplication of these two parameters (absolute values ranging from 0 to 400). For certain analyses, the level of expression in samples was scored as: negative or low, intermediate, and high, which corresponded to Hirsch values of 0–200, 201–300, and 301–400, respectively.

We also tested the following antibodies: Ki67 (clone MiB-1, DakoCytomation, Glostrup, Denmark; 1/200^e^), p53 (Dako Cytomation; 1/50^e^), Olig2 (Immuno-Biological Laboratories, Gunma, Japan; 1/200^e^), and glial fibrillar acidic protein (Dako Cytomation; 1/1600^e^). The percentages of cells labeled with these antibodies were determined by studying at least five hundred cells for each antibody in tumor areas of highest positivity.

### Total RNA extraction

Tumor tissue (30 mg) was incubated with 1 ml Qiazol^®^ solution (Qiagen, Courtaboeuf, France) and CK14 ceramic beads (Ozyme) and then pulverized two times for 40 sec each at 6500 rpm in a Precellys 24 homogenizer (Bertin Technologies). Homogenized tissues were then lysed and RNA purification was performed according to the manufacturer’s protocol (RNeasy Lipid Tissue Mini kit, Qiagen). RNA concentration and purity was estimated by spectrophotometry (NanoDrop ND1000, Labtech, France). RNA quality was assessed by capillary electrophoresis (Bioanalyzer 2100, Agilent Technologies) and only RNA with an Integrity Number (R.I.N) >6 was used for analysis.

### Quantitative and qualitative RT-PCR

Complementary DNA (cDNA) was synthesized from 2 μg of total RNA using the Transcriptor First Strand cDNA Synthesis^®^ kit (Roche) and hexamer primers, according to the manufacturer’s protocol. For PCR, primers were designed using Amplify 1.2 or Primer 3 (http://fokker.wi.mit.edu/primer3/input.html/) software and their specificity was determined by BLASTn in the NCBI database (http://www.ncbi.nlm.nih.gov/). Primer characteristics are listed in Table I. Amplicon size and specificity were initially determined by 4% NuSieve agarose gel electrophoresis. Quantitative PCR [EGFRv1, -v2, -v3, -v4 and hypoxanthine phosphoribosyl transferase (HPRT)] and qualitative PCR (EGFRvIII) were performed on a Rotor Gene thermocycler (Corbett Research) using the Light Cycler Fast Start DNA Master SYBR Green I kit (Roche). All targets were amplified twice in duplicate in the presence of 3 mM MgCl_2_ and 0.5 μM primers. Relative quantification of mRNA content was performed using the ΔΔCt method [(Ct_sample_-Ct_calibrator_)_interest gene_ - (Ct_sample_-Ct_calibrator_)_reference gene_], modified according to Pfaffl (29), with efficiency correction by Rotor Gene software. mRNA content was expressed in relative arbitrary units (R.A.U.).

### Fluorescent in situ hybridization

*EGFR* gene amplification and 1p36 and 19q13 losses were analyzed by double fluorescent *in situ* hybridization with the ‘LSI *EGFR* SpectrumOrange/CEP 7 SpectrumGreen Probe’, or with the ‘LSI 1p36 spectrum orange/LSI 1q25 spectrum green probe’ and the ‘LSI 19q13 spectrum orange/LSI 19p13 spectrum green probe’ (Abbott Molecular Inc., IL, USA) kits, respectively. They were investigated on consecutive paraffin sections from the same blocks used in immunohistochemical analyses. This technique was a modification of the method previously described (28): briefly, 4 μm paraffin sections were incubated 16 h at 56°C, submitted to deparaffinising, digested with pepsin (Abbott Molecular Inc.) at 37°C during 45 min and dehydrated in successive ethanol baths. Slides were incubated with 10 μl of each probe for 5 min at 73°C to denature DNA and 16 h at 37°C to ensure hybridization. Sections were washed in 2X SSC/0.3% NP40 solution, once for 1 min at room temperature, once for 2 min at 73°C, and dehydrated in successive ethanol baths. Counterstaining and microscopic observation of *EGFR* amplification were performed as previously described (28). *EGFR* gene amplification was considered to have occurred if more than 10% of the cells analyzed produced a red (corresponding to the *EGFR*-specific probe) to green (centromeric region of chromosome 7) signal ratio ≥2, as recommended previously (30,31). Eight sequential focus stacks with 0.3 μm were acquired and then integrated into a single image in order to reduce thickness related artifacts. Preparations were considered as valid when more of 80% of the cells showed bright signals.

### Statistical analyses

StatView^®^ 5.0 software (SAS Institute, Inc., Cary, NC, USA) was used for statistical analyses. Fisher’s exact test was used to assess differences between nominal variables. Means were compared with the non-parametric Mann-Whitney test for pairs of variables and with the Kruskall-Wallis tests for comparisons of more than two variables. Correlation Spearman test was used to compare quantitative variables. Overall survival (OS) and progression-free survival (PFS) were analyzed by Kaplan-Meier and median OS or PFS medians were compared with the non-parametric log-rank or Breslow tests. Results for which p<0.05 were considered to be statistically significant.

## Results

### Patient and tumor characteristics

Patient characteristics are summarized in Table II. For the series, median follow-up was 23.3 (0.5–240) months. Ki67 labeling index, Olig2 and p53 protein expression, and presence or absence of a 1p36–19q13 loss were consistent with histological typing. Thus, oligodendrogliomas were characterized by a 1p36/19q13 deletion, stronger olig2 and weaker p53 expressions compared to other tumor types (Table III).

### Immunohistochemical detection of EGFR isoforms and EGFRvIII mutant

**Figure 2:**
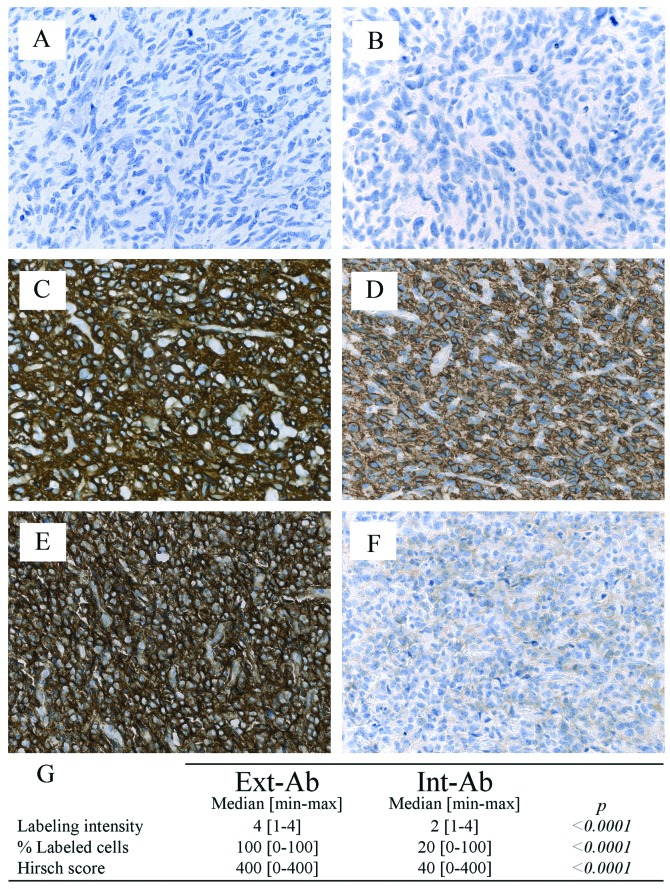
Immunohistochemical staining. Absence of labeling with both Ext-Ab (A) and Int-Ab (B) in a glioblastoma. Strong EGFR expression with both Ext-Ab (C) and Int-Ab (D) in a glioblastoma. Discordant staining: strong with the Ext-Ab (E) and very weak with the Int-Ab (F) in a grade III oligodendroglioma. (G), table of statistical analysis.

The percentage of gliomas stained by Ext-Ab was 98% (44/45), whereas only 78% (35/45) of the gliomas were stained by Int-Ab. The Ext-Ab and Int-Ab antibodies generated very different staining patterns in the gliomas (Fig. 2A-F). Glioma staining by Int-Ab was significantly lower than that of Ext-Ab in terms of intensity, percentage of positive cells and Hirsch score (p<0.0001; Fig. 2G).

Neither antibody detected any significant differences in EGFR staining with respect to patient sex or age (data not shown). Staining intensities, percentages of labeled cells and Hirsch scores obtained with Ext-Ab did not significantly differ according to histological types. In contrast, Int-Ab scores were significantly higher in glioblastomas, astrocytomas or oligodendrogliomas compared to oligoastrocytomas (Table IV). In non-tumor tissue, we found no or very weak EGFR expression whatever the antibody used.

### Quantitation of EGFR variants 1, 2, 3, 4 and EGFRvIII mRNAs

**Figure 3:**
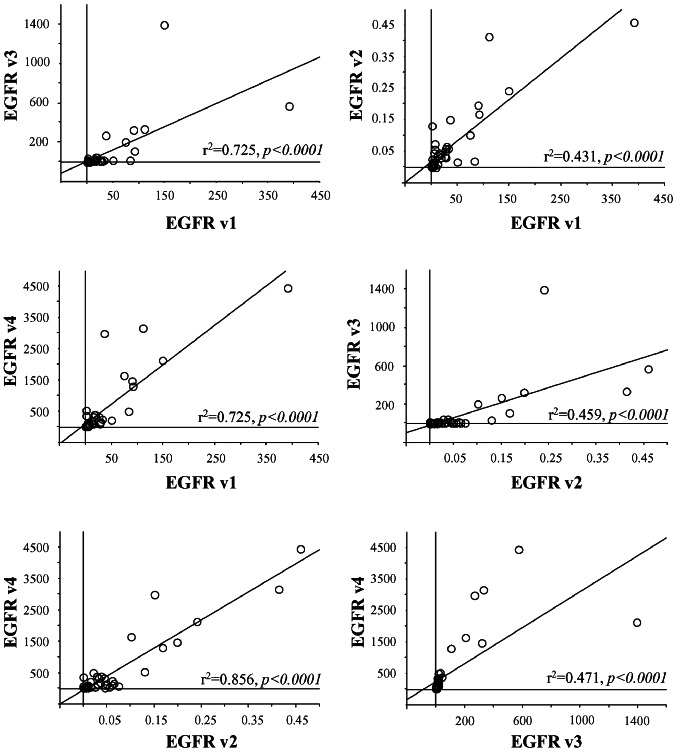
Correlation for EGFRv1+vIII, -v2, -v3 and -v4 mRNA levels.

EGFR mRNA levels varied widely among the tumor samples. Median R.A.U. values were 7.3 (0.4–390.2) for EGFRv1+vIII mRNA, 0.02 (0–0.5) for EGFRv2 mRNA, 6.2 (0.1–1396.8) for EGFRv3 mRNA and 94.6 (2.1–4445.2) for EGFRv4 mRNA. Straight correlations were found for all comparisons between EGFRv1+vIII, -v2, -v3 and -v4 mRNA levels (p<0.0001 for all, Fig. 3). EGFRvIII mutant expression was qualitatively detected in 26% (12/47) of the gliomas. In non-tumor tissue mRNA variant were very weakly expressed with 0.7 R.A.U. for EGFRv1+vIII and EGFRv3, 6.6 R.A.U. for EGFRv4 and no EGFRvIII and EGFRv2 expression.

### Association of EGFR variant and EGFRvIII mRNA expression with other parameters

**Figure 4:**
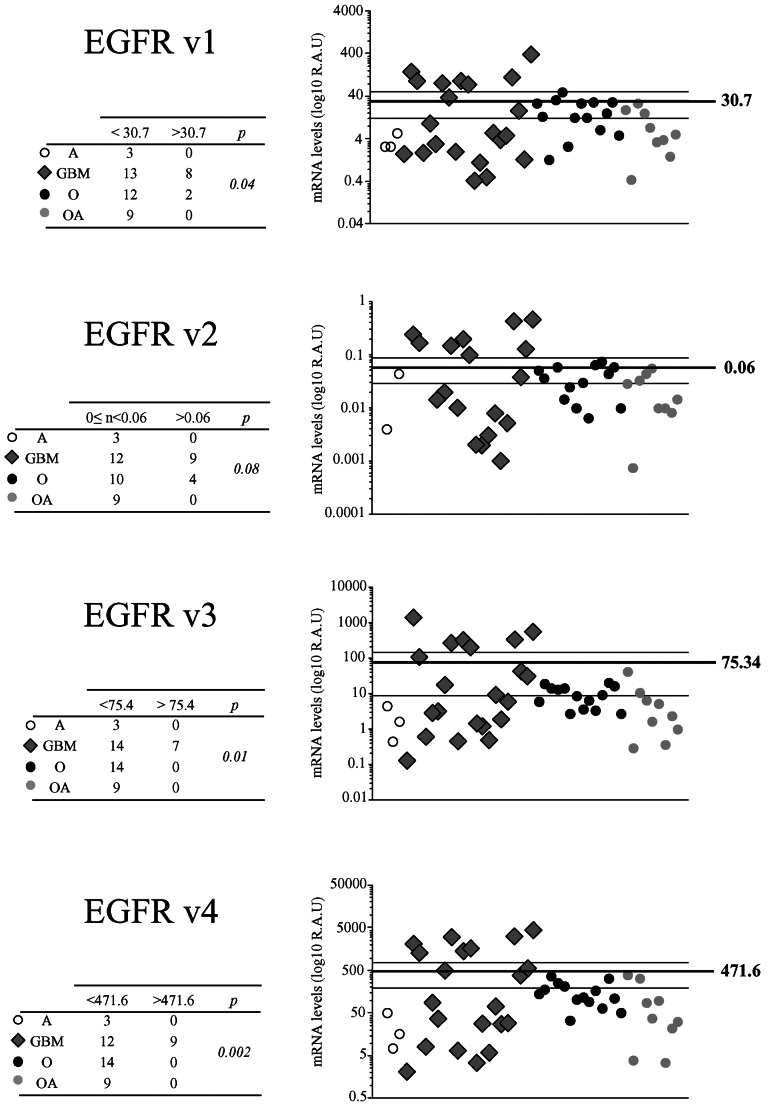
Relation between EGFR variant mRNA levels and histological types. EGFRv1+vIII, -v2, -v3 and -v4 mRNA levels were expressed in Log10 relative arbitrary unit (Log10 R.A.U). Mean value was taken as a cut-off to determine the number of tumors under (< mean) and above (> mean) it.

**Figure 5:**
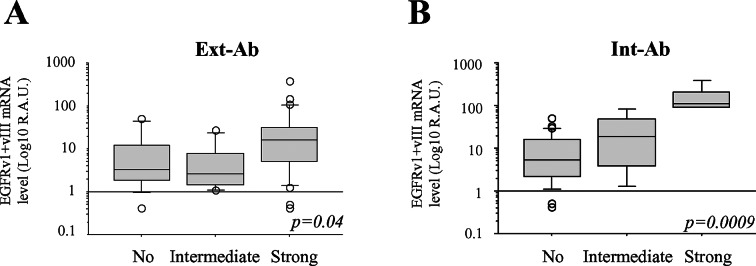
Relation between EGFRv1+vIII mRNA levels and immunohistochemical data. Ext-Ab (A) and Int-Ab (B) labeling were expressed as no vs. intermediate vs. strong Hirsch score.

EGFRv1+vIII, -v2, -v3, and -v4 mRNA levels were not influenced by patient sex and age (data not shown). EGFRv1+vIII, -v3 and -v4 mRNA levels were higher in glioblastomas than in other tumor types when mean values were taken as a cut-off (p=0.04, 0.01 and 0.002, respectively) (Fig. 4). EGFRvIII mRNA was significantly associated with histological type, it was found in one astrocytoma, ten glioblastomas and one oligoastrocytoma, but in none oligodendroglioma (p=0.01, data not shown). We also observed that EGFRv1+vIII mRNA levels more straightly correlated with Int-Ab staining than with Ext-Ab staining (Fig. 5).

### EGFR gene amplification in gliomas

We detected *EGFR* gene amplification in 8 out of the 20 glioblastomas, but not in the other glioma types (p=0.007, Table V). Glioblastomas with *EGFR* gene amplification expressed significantly stronger EGFRv1, -v2, -v3 and -v4 mRNA levels than gliomas with no *EGFR* amplification (Table V). The presence of mutant EGFRvIII mRNA was significantly associated with EGFR amplification (p<0.0001).

**Figure 6:**
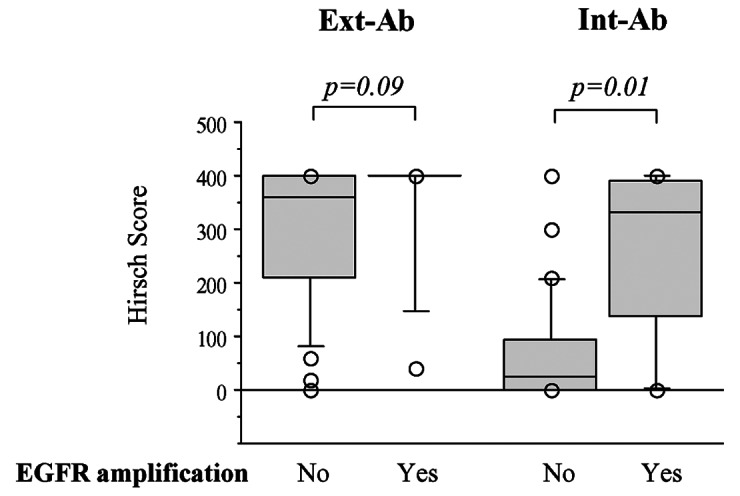
Association between EGFR gene amplification and immunohistochemical staining. Ext-Ab (A) and Int-Ab (B) staining expressed as Hirsch scores was compared in tumors exhibiting or lacking EGFR gene amplification.

Weak Ext-Ab staining was more closely associated with the absence of *EGFR* gene amplification than with its presence (p=0.09), whereas strong Int-Ab staining was significantly associated with gene amplification (p=0.01; Fig. 6).

### Prognostic values

**Figure 7:**
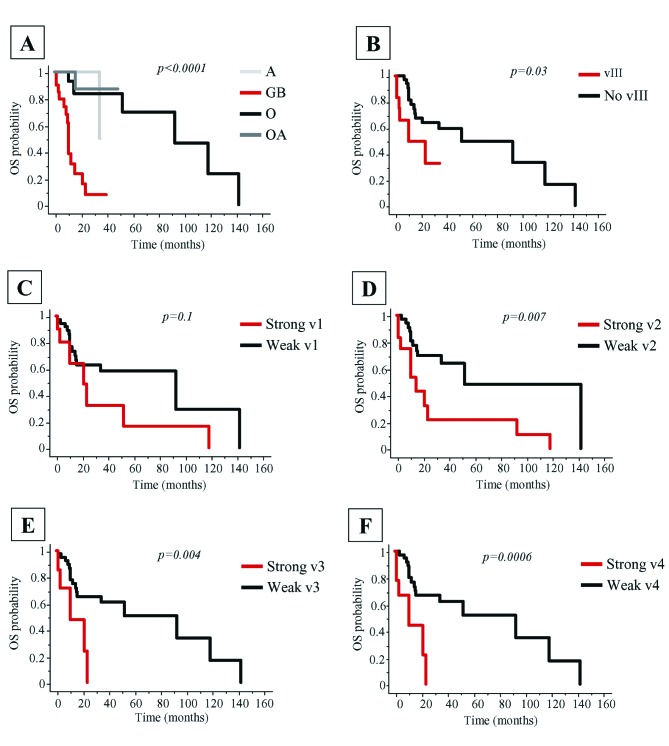
Patient overall survival (OS). Differences in OS according to histological types (A), mutant EGFRvIII expression (B), EGFRv1+vIII (C), -v2 (D), -v3 (E) and -v4 (F) mRNA levels were assessed by log-rank test.

PFS and OS were significantly shorter for patients diagnosed with glioblastoma and astrocytoma than for those with oligodendroglioma and oligoastrocytoma (p<0.0001 for both) (Table VI, Fig. 7A). OS and PFS were better for patients with tumors showing a 1p36–19q13 loss and an absence of *EGFR* amplification (Table VI).

No/intermediate or strong tumor labeling with EGFR Ext-Ab did not influence OS and PFS times whereas no/intermediate labeling with EGFR Int-Ab was associated with longer OS and PFS (Table VI). In addition, PFS and OS were longer when gliomas expressed weak EGFRv1+vIII, -v2, v3, or -v4 mRNA levels and showed no mutant EGFRvIII mRNA expression (Table VI, Fig. 7B-F).

In glioblastomas (data not shown), PFS was significantly better for patients with no *EGFR* amplification (5.4 vs. 8.4 months, p=0.01), no expression of EGFRvIII mutant mRNA (3.7 vs. 8.4 months, p=0.04), or weak EGFRv2 (3.3 vs. 5.6 months, p=0.04) or -v4 mRNA levels (8.4 vs. 4.7 months, p=0.05). OS did not change according to these parameters.

## Discussion

Based on the present results, we report that diffuse gliomas expressed truncated EGFR protein isoforms, based on: i) immunohistochemical data and ii) EGFRv1, -v2, -v3, -v4 variants and EGFRvIII mutant mRNA detection.

Immunohistochemical results varied according to the antibody used and favored the hypothesis of an expression of the truncated isoforms. The stronger EGFR staining obtained with Ext-Ab reflected their expression since, in addition to functional EGFR and the EGFRvIII mutant targeted by both antibodies, Ext-Ab also recognized the truncated EGFR isoforms b, c, and d whereas Int-Ab did not. The detection of truncated isoforms, depending on the antibody used, could explain some of the discrepancies found in literature regarding EGFR expression in gliomas (9–15). In our series, glioblastomas and oligodendrogliomas were strongly labeled by both Ext-Ab and Int-Ab, whereas oligoastrocytomas were moderately or strongly labeled by Ext-Ab but weakly by Int-Ab. Thus, in combination with other markers such as Olig2, p53 or 1p19q deletion, the study of EGFR expression might be useful to further characterize the diffuse gliomas (32–36).

The transcriptomic analysis showed that alternatively spliced EGFRv2, -v3 and -v4 transcript variants, encoding EGFR isoforms b, c and d, respectively, were expressed in addition to the EGFRv1 and EGFRvIII mutant mRNAs. In accordance with immunohistochemistry, detection of EGFRv1+vIII mRNA associated more closely with Int-Ab staining than with Ext-Ab staining. However, we also found an association between Int-Ab staining and EGFRv2, -v3 and -v4 transcript expressions, although Int-Ab did not detect the isoform they encode. This association is probably the consequence of the strong link existing between the expressions of each EGFR transcript (Fig. 3). Nevertheless, alternatively spliced EGFR transcript variants and EGFRvIII mRNA were produced at different levels according to the histological type of glioma. The glioblastomas had a peculiar profile. They expressed the highest levels of EGFRv3 and -v4 mRNA transcripts and, in addition, EGFRvIII mRNA expression was related to this tumor type.

*EGFR* gene amplification was detected in eight glioblastomas. As previously reported (28), weak Ext-Ab staining was associated with the lack of *EGFR* amplification. In contrast, Int-Ab staining intensity directly correlated with *EGFR* gene amplification, as already shown (37). This suggests that *EGFR* gene amplification is tightly associated with high expression of EGFR receptor isoforms that contain the intracellular tyrosine kinase domain, i.e., EGFR isoform a and the EGFRvIII mutant. Regarding the relationships with the prognosis in our series, the histological type (astrocytoma and glioblastoma), a strong staining with Int-Ab and the presence of *EGFR* amplification and of mutant vIII were associated with shorter PFS and OS times. High levels of EGFRv2, -v3 and -v4 transcript expression were also related to a shortened OS and PFS.

Our data tend to indicate that the role of EGFR pathway in glioblastoma oncogenesis is more complex than expected. In our series, in addition to the known molecular alterations i.e., *EGFR* gene amplification and expression of vIII mutant, we observed a strong staining with Int-Ab and high levels of EGFR mRNA variants. Actually, the functional roles of the truncated EGFR isoforms are poorly known, particularly *in vivo. In vitro*, it has been described that soluble isoforms may regulate EGFR signaling in normal and tumor cells (26,38). Paradoxically, these isoforms have been shown to decrease cellular proliferation (22,23). The known mechanisms responsible for growth inhibition include competitive binding of ligands to soluble isoforms and formation of inactive heterodimers which inhibit the formation of holoreceptor dimers and/or intracellular kinase activity (24–26).

The presence of truncated EGFR isoforms in adult infiltrating gliomas must be considered in therapeutic management. The interactions between truncated EGFR isoforms and EGFR-targeted therapeutics are not well understood. The presence of non-functional receptors could contribute to the failure of therapeutics which target the EGFR extracellular domain (39). Conversely, it has been reported that the presence of truncated EGFR isoforms may be predictive of the therapeutic response to gefitinib in endometrious adenocarcinomas (40). Lafky and coworkers speculated that the soluble vascular endothelial growth factor receptor (sVEGFR) represents a paradigm for understanding the function and potential application of EGFR isoforms as novel therapeutic molecules (41). Soluble VEGFR isoforms have been presented as effective therapeutic molecules (42) and a similar application for certain EGFR truncated isoforms may be possible.

To our knowledge, this is the first report that gliomas express EGFR transcripts other than EGFRv1 mRNA, which encodes the full-length and functional EGFR isoform a. The role of EGFR isoforms in glioma pathogenesis remains to be clarified, but their expression makes them potential targets of future diagnostic and therapeutic strategies.

## Figures and Tables

**Table I:** Characteristics of the primer used for quantitative and qualitative RT-PCR.

Target	Primer	Location	Sequence 5′-3′	Amplicon size (bp)	Primer temperature (°C)
EGFR variant 1	Forward	Exon 29–30	CTCCCAGTGCCTGAATACATA	83	58
	Reverse	Exon 30	GGCTGATTGTGATAGACAGGA		
EGFR variant 2	Forward	Exon 15	TGCACCTACGGGTCCTAAT	97	58
	Reverse	Exon 16	TGAAGCAAAGGGAGAAATTG		
EGFR variant 3	Forward	Exon 10	AAGGAAATCACAGGTTTGAGC	99	58
	Reverse	Exon 10bis	TCCAAGGGAACAGGAAATATG		
EGFR variant 4	Forward	Exon 15	CTACGGGCCAGGAAATGAG	86	62
	Reverse	Exon 17	CGCTGCCATCATTACTTTGA		
HPRT	Forward	Exon 6	CTTTCCTTGGTCAGGCAGTA	90	58
	Reverse	Exon 7	TGGCTTATATCCAACACTTCG		
EGFRvIII	Forward	Exon 1	GCTCTGGAGGAAAAGAAAGGTAAT	90	62
	Reverse	Exon 8	TCCTCCATCTCATAGCTGTCG		

**Table II:** Demographic, pathological and clinical features.

	No.	Grade	Sex (male/female)	Age (median)	Tumor status (primary/recurrent)	Radiotherapy (yes/no)	Chemotherapy (yes/no)
All	47		18/19	50.6	36/11	42/5	37/10
A	3	1 II, 2 III	1/2	51.3	2/1	2/1	2/1
GBM	21	21 IV	10/11	59.7	19/2	20/1	18/3
O	14	5 II, 9 III	3/11	48.1	10/4	13/1	13/1
OA	9	6 II, 3 III	4/5	43.2	5/4	7/2	4/5

A, astrocytoma; GBM, glioblastoma; O, oligodendroglioma; OA, oligoastrocytoma.

**Table III:** Molecular characterization of glioma histological types.

	Ki67 (n=45)		p53 (n=45)		Olig2 (n=45)		1p36-19q13 loss (n=46)		
				
	% Mean ± SD	p-value	% Mean ± SD	p-value	% Mean ± SD	p-value	Yes	No	p-value
All patients	19±14		46.3±31.1		58.6±23.9		17	29	
Histological type									
A	13.7±10	0.06	80±10	0.0004	51.7±27.5	0.04	0	3	<0.0001
GBM	26.9±13.5		49.7±28.3		48.7±25.8		2	18	
O	14.8±19		20.6±18.4		71.8±18.3		14	0	
OA	10.8±11.2		67.8±28.5		61.1±19		1	8	

A, astrocytoma; GBM, glioblastoma; O, oligodendroglioma; OA, oligoastrocytoma.

**Table IV:** Association between Ext-Ab and Int-Ab labeling and pathological parameters.

		Ext-Ab						Int-Ab					
			
	No.	Labeling intensity	p-value	Percentage of labeled cells	p-value	Hirsch score	p-value	Labeling intensity	p-value	Percentage of labeled cells	p-value	Hirsch score	p-value
All	45	4 [1–4]		100 [0–100]		400 [0–400]		2 [1–4]		20 [0–100]		40 [0–400]	
Histological type													
A	3	4 [3–4]	0.73	70 [20–90]	0.33	360 [60–400]	0.69	2 [2–3]	0.006	30 [5–40]	0.01	60 [10–120]	0.01
GBM	19	4 [2–4]		100 [10–100]		400 [20–400]		3 [1–4]		30 [0–100]		90 [0–400]	
O	14	4 [1–4]		100 [0–100]		380 [0–400]		2 [1–3]		45 [0–70]		90 [0–210]	
OA	9	4 [2–4]		100 [20–100]		300 [80–400]		1 [1–2]		0 [0–10]		0 [0–20]	

Ext-Ab, antibody targeting the extracellular domain; Int-Ab, antibody targeting the intracellular domain; A, astrocytoma; GBM, glioblastoma; O, oligodendroglioma; OA, oligoastrocytoma.

**Table V:** *EGFR* amplification.

	*EGFR* amplification		
	
	No.	Yes	p-value
Histological type			
A	3	0	0.007
GBM	12	8	
O	13	0	
OA	9	0	
EGFRv1+vIII mRNA			
< mean	34	2	0.0001
> mean	3	6	
EGFRv2 mRNA			
< mean	32	2	0.001
> mean	5	6	
EGFRv3 mRNA			
< mean	36	1	<0.0001
> mean	3	5	
EGFRv4 mRNA			
< mean	36	1	<0.0001
> mean	1	7	
EGFRvIII mRNA			
Present	4	7	<0.0001
Absent	33	1	

A, astrocytoma; GBM, glioblastoma; O, oligodendroglioma; OA, oligoastrocytoma.

**Table VI:** PFS and OS according to histological and molecular parameters.

	PFS		OS	
		
	Median (months)	p-value	Median (months)	p-value
Histological types				
A	16.4	<0.0001	34.9	<0.0001
GBM	5.4		10.9	
O	45.1		93.3	
OA	41		nr	
EGFR amplification				
Yes	4.7	<0.0001	8.8	0.0003
No	21.6		93.3	
1p36-19q13 loss				
Yes	21.9	0.04	93.3	0.09
No	16.4		24	
Ext-Ab				
No/intermediate	22.7	0.25	52.8	0.7
Strong	16.4		93.3	
Int-Ab				
No/intermediate	21.4	0.006	93.3	0.07
Strong	4.7		21.6	
EGFRv1 mRNA				
Weak	21	0.02^a^	93.3	0.1
Strong	5.4		21.6	
EGFRv2 mRNA				
Weak	21.4	0.01^a^	52.8	0.007
Strong	9		15.1	
EGFRv3 mRNA				
Weak	21	0.0007	93.3	0.004
Strong	4.7		10.9	
EGFRv4 mRNA				
Weak	21.4	<0.0001	93.3	0.0006
Strong	4.7		10.9	
EGFRvIII mRNA				
No	21.4	0.0007	52.8	0.03
Yes	4.7		10.9	

a The difference was not significant using log-rank test but was significant with Breslow-Gehan-Wilcoxon test.

OS, overall survival; PFS, progression-free survival; Nr, not reached; A, astrocytoma; GBM, glioblastoma; O, oligodendroglioma; OA, oligoastrocytoma.
